# A Single Dose Versus Two Doses of Tranexamic Acid for Extracapsular Hip Fractures

**DOI:** 10.7759/cureus.21239

**Published:** 2022-01-14

**Authors:** Max Jiganti, Olivia Pipitone, Justin Than, Richard Stanley, Angela Passanise, Jacqueline Krumrey

**Affiliations:** 1 Orthopedics, Good Samaritan Regional Medical Center, Corvallis, USA; 2 Graduate Medical Education - Biostatistics, Good Samaritan Regional Medical Center, Corvallis, USA; 3 Orthopedics, The Corvallis Clinic, Corvallis, USA; 4 Orthopedics and Traumatology, Good Samaritan Regional Medical Center, Corvallis, USA

**Keywords:** basicervical femoral neck, extracapsular hip fracture, subtrochanteric, intertrochanteric, tranexamic acid

## Abstract

Objective

In this study, we aimed to compare the effectiveness of one dose of tranexamic acid (TXA) at the time of hospital admission versus two doses of TXA (one at the time of hospital admission and another dose intraoperatively) in reducing perioperative total blood loss in patients with extracapsular hip fractures.

Methods

This retrospective cohort study included 80 patients from a single institution who underwent surgical fixation for extracapsular hip fractures. Forty patients received a single dose of 1 gram of TXA at the time of hospital admission (per standardized protocol of an ongoing research study at the time), and 40 patients received the same dose of TXA on hospital admission as well as a second dose of 1 gram of TXA intraoperatively at the time of incision (per standard practice change following the completion of the research study). The primary study outcome of interest was total blood loss, which was calculated by estimating blood volume via Nadler’s formula followed by calculating the total blood loss with the hemoglobin dilution method. Secondary outcomes included blood transfusion rates, hospital length of stay (LOS), and 30-day mortality.

Results

Patient gender, age, the American Society of Anesthesiologists (ASA) score, procedure length, fracture type, hardware type, and hemoglobin on hospital arrival were similar across the study groups (all p>0.05), though the twice-dosed group had a higher average BMI (26.4 kg/m^2^ vs. 24 kg/m^2^, p=0.04). When adjusting for BMI, the twice-dosed group was estimated to have a slightly larger but non-significant difference in total blood loss (115-ml difference, 95% CI: 158.2-389.3, p=0.40) compared to the single-dose TXA group. More patients in the twice-dosed group required blood transfusion compared to the single-dose TXA group, though this was not statistically significant (30.0% vs. 17.5%, adjusted OR=1.64, 95% CI: 0.55-5.12, p=0.38). The distribution of hospital LOS and 30-day mortality rates were similar across the groups (p=0.13 and p>0.99).

Conclusion

In the setting of surgically treated extracapsular hip fractures, patients who received one dose of TXA at the time of hospital admission and a second intraoperative dose of TXA did not demonstrate significant differences in total blood loss or a need for blood transfusion compared to patients who only received a single dose of TXA at the time of hospital admission.

## Introduction

Over 300,000 hip fractures occur annually in the US in individuals aged >65 years, with this number expected to double by the year 2050 [1]. Blood loss in hip fractures is a common complication that may lead to perioperative anemia, necessitating allogeneic blood transfusion [2]. Patients with extracapsular hip fractures, as opposed to intracapsular hip fractures, are associated with increased postoperative blood loss as well as higher rates of blood transfusion [3,4].

Tranexamic acid (TXA) is an effective medication utilized to help minimize blood loss after hip fractures and during surgery. It acts to stabilize clot formation by effectively blocking lysine-binding sites of plasminogen, which inhibits fibrinolytic activity and fibrin degradation [5]. TXA is commonly utilized in surgical treatments of extracapsular hip fractures (basicervical femoral neck, intertrochanteric femur, and subtrochanteric femur fractures), and it has been shown to effectively reduce intraoperative blood loss without increasing the thromboembolism risk [6-8]. TXA has been shown to be effective with a single dose either at admission or intraoperatively [7,8]. Two doses of TXA given at different intervals, one intraoperatively followed by another dose postoperatively, have also been shown to be effective [6]. Although studies have shown that intervention with TXA is safe and helps to minimize blood loss, there is no consensus regarding the most effective timing or frequency of TXA intervention.

Our study aims to compare the effectiveness of one dose of TXA at the time of hospital admission versus two doses of TXA (one at the time of hospital admission and another dose intraoperatively) in decreasing perioperative total blood loss in patients with extracapsular hip fractures. To the best of our knowledge, this is the first study to compare these two protocols. Our hypothesis was that giving TXA twice (once on admission and again intraoperatively) would reduce total blood loss and the need for blood transfusions when compared to giving only one dose of TXA at the time of admission.

## Materials and methods

Study design

This retrospective cohort study was reviewed by the Samaritan Health Services Regional Institutional Review Board (IRB) and approved as exempt (approval number: IRB20-102). Data were collected from electronic medical records of patients over the age of 50 years presenting to the hospital between October 2015 and May 2021 with closed intertrochanteric, subtrochanteric, or basicervical femur fractures. Surgeries were consistently performed by resident physicians with attending oversight. Data related to patient age, gender, BMI, American Society of Anesthesiologists (ASA) score, procedure length, fracture type, hardware type, and hemoglobin levels on hospital arrival, postoperative day (POD) one, and POD three or four were extracted from electronic medical records. Operative notes were examined to identify intraoperative blood loss. The number of blood transfusions that each patient had received was tracked from hospital arrival to POD five or the date of discharge, whichever came first. Nadler’s formula was utilized to estimate blood volume according to gender, height, and weight [9]. The hemoglobin dilution method was then used to calculate total blood loss, which is notably higher than intraoperative blood loss alone [7].

Inclusion and exclusion criteria

At this institution, a randomized clinical trial had taken place from October 2015 to January 2019 to compare the effect of 1 gram of intravenous TXA with that of normal saline on hospital arrival in patients undergoing surgical treatment for extracapsular femur fractures [10]. After the conclusion of that trial in January 2019, our institution had adopted a new hip fracture treatment protocol, which involves giving 1 gram of intravenous TXA at the time of hospital admission and another dose of 1 gram of intravenous TXA intraoperatively at the time of incision. For the present study, we included patients who met our inclusion criteria and had received 1 gram of TXA at admission in the previously concluded trial. We then identified patients who met our inclusion criteria and were treated under the new hip fracture treatment protocol, meaning they had received two doses of TXA: 1 gram at admission and 1 gram intraoperatively. Patients were excluded if they were on anticoagulation before surgery, had renal impairment [defined as glomerular filtration rate (GFR) <30 or creatinine (Cr) >1.5], had inadequate medical record data to calculate estimated total blood loss, had more than one surgery during their admission, received anything other than aspirin alone postoperatively for deep vein thrombosis (DVT) prophylaxis, or if they had multiple fractures.

Statistical analysis

Statistical analysis was performed using the R software version 3.6.1. An a priori power analysis was conducted for our primary outcome of interest, estimated total blood loss. Assuming a pooled standard deviation of 537 ml based on data collected from the previous randomized clinical trial conducted at our institution, we estimated that a two-sample t-test comparing total estimated blood loss across study groups would be adequately powered (80%) at an alpha of 0.05 and a sample size of 40 patients in each study group to detect a difference between groups in terms of estimated total blood loss of 341 ml or higher.

Two-sample t-tests were used to compare patient age, BMI, and hemoglobin level on hospital arrival across study groups. Chi-squared tests were used to compare patient gender and fracture type. Due to small cell sizes, Fisher’s exact tests were used to compare ASA scores and hardware types. A two-sample t-test was used to compare total blood loss across study groups. Because BMI was found to be significantly higher in patients who received two doses of TXA, a linear regression model was also used to explore total blood loss across study groups when adjusting for the effect of BMI. An unadjusted OR was calculated for exploring the odds of needing a blood transfusion by the study group. To account for differences between study groups in BMI, a logistic regression model was also used to explore the odds of needing a blood transfusion by the study group when adjusting for the effect of BMI. A Fisher’s exact test was used to compare hospital length of stay (LOS) and 30-day mortality.

## Results

Patient gender, age, ASA score, procedure length, fracture type, hardware type, and hemoglobin on hospital arrival were similar across study groups (all p>0.05), as shown in Table 1; however, the twice-dosed group had a significantly higher average BMI (26.4 kg/m^2^ vs. 24 kg/m^2^, p=0.04).

**Table 1:** Participant characteristics P-values: two-sample t-test for age, BMI, hemoglobin level on hospital arrival. Chi-squared test for gender and fracture type. Fisher’s exact test for ASA, procedure length, and hardware type TXA: tranexamic acid; SD: standard deviation; ASA: American Society of Anesthesiologists

Variables	Preop TXA only (n=40)	Preop with intraop TXA (n=40)	P-value
Female, % (n)	72.5% (29)	72.5% (29)	>0.99
Age in years, mean (SD)	82.2 (10.2)	80.1 (9.6)	0.35
BMI, kg/m^2^, mean (SD)	24.0 (4.3)	26.4 (5.6)	0.04
ASA score, % (n)			
1	2.5% (1)	0.0% (0)	0.25
2	30.0% (12)	17.5% (7)	
3	62.5% (25)	70.0% (28)	
4	5.0% (2)	12.5% (5)	
Procedure duration, % (n)			
<1 hour	52.5% (21)	32.5% (13)	0.19
1-2 hours	40.0% (16)	57.5% (23)	
>2 hours	7.5% (3)	10.0% (4)	
Fracture type, % (n)			
Intertrochanteric	85.0% (34)	82.5% (33)	>0.99
Subtrochanteric	15.0% (6)	17.5% (7)	
Hardware type, % (n)			
Cephalomedullary nail	97.5% (39)	100.0% (40)	>0.99
Dynamic hip screw	2.5% (1)	0.0% (0)	
Hemoglobin on hospital arrival, g/dl, mean (SD)	12.6 (1.6)	12.2 (1.4)	0.35

**Figure 1:**
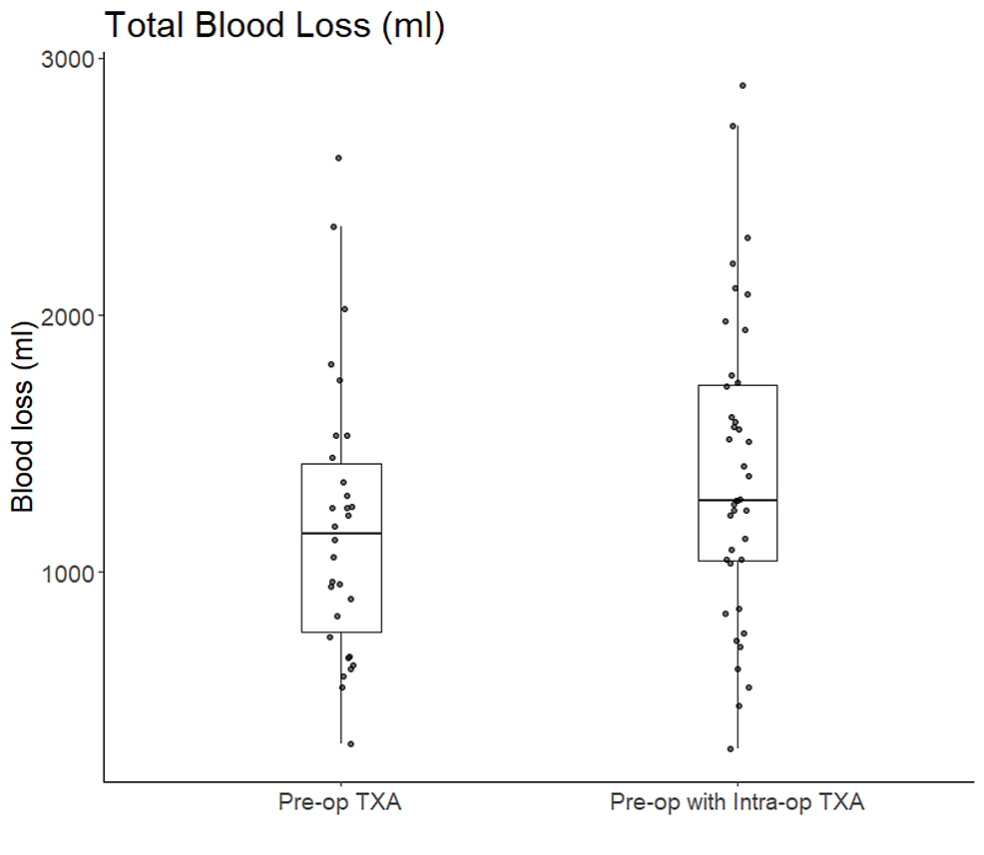
Distribution of total blood loss TXA: tranexamic acid

Figure 1 shows the distribution of total blood loss across the two study groups. In the single-dose TXA group, 10 patients did not have adequate hemoglobin levels recorded during their hospital stay to allow for the calculation of total blood loss via the hemoglobin dilution method and were therefore excluded from the analysis of total blood loss. The average total blood loss was higher in the twice-dosed TXA group compared to the single-dose TXA group (Table 2), but this difference was not statistically significant in unadjusted analyses or in analyses adjusting for the effect of BMI (unadjusted average difference: 208.4 ml, 95% CI: -482.0-65.1, p=0.13; adjusted average difference: 115.5 ml, 95% CI: -158.2-389.3, p=0.40). More patients required blood transfusion in the twice-dosed group compared to the single-dose group, though there was not a significant difference in the odds of needing a blood transfusion, whether or not BMI was adjusted for (unadjusted OR=2.02, 95% CI: 0.70-5.83, p=0.19; adjusted OR=1.64, 95% CI: 0.55-5.12, p=0.38).

A higher proportion of patients in the twice-dosed group had a hospital stay of seven or more days (32.5% vs. 15.0%); however, the distribution of LOS did not significantly differ across the study groups; 30-day mortality rates were also similar between the groups (Table 2).

**Table 2:** Participant outcomes P-values: Chi-squared test for received blood transfusion, two-sample t-test for total blood loss. Fisher’s exact test for the length of hospital stay and 30-day mortality. For total blood loss, data was only available for 30 out of 40 patients in the single-dose TXA group TXA: tranexamic acid; SD: standard deviation

Variables	Single-dose TXA (n=40)	Twice-dosed TXA (n=40)	P-value
Received blood transfusion, % (n)	17.5% (7)	30.0% (12)	0.29
Total blood loss, ml (SD)	1181.8 (537.0)	1390.2 (589.3)	0.13
Length of hospital stay, % (n)			
1-3 days	22.5% (9)	10.0% (4)	0.13
4-6 days	62.5% (25)	57.5% (23)	
7+ days	15.0% (6)	32.5% (13)	
30-day mortality, % (n)	5.0% (2)	2.5% (1)	>0.99

## Discussion

TXA plays a vital role in reducing blood loss and the need for blood transfusions in patients undergoing surgery for hip fractures [11-13]. TXA has also been shown to significantly diminish hidden blood loss in the setting of hip fractures, which occurs outside of surgery, without an increase in thromboembolic risk [6,7,14-16]. Due to the clear benefits of TXA in such cases, optimal dosing may provide further advantages for these patients.

Our results revealed no significant difference in total blood loss or transfusions between administering a single dose of TXA upon hospital arrival alone versus administering TXA both upon hospital arrival and again intraoperatively in patients with extracapsular hip fractures. Contrary to our initial hypothesis, we found no benefit in the addition of an intraoperative dose of TXA to the dose on admission. In fact, we observed an increased risk of requiring a blood transfusion in the twice-dosed group, which was not anticipated, though this was not statistically significant. It is unlikely that an additional dose of TXA has a paradoxical effect, and it more likely indicates that there was no added benefit from an additional dose.

To our knowledge, there is currently no study in the literature on the impact of TXA dosing on estimated total blood loss in patients with extracapsular hip fractures. A recent level III retrospective study evaluated the difference between single- and twice-dosing of TXA in patients undergoing total hip or total knee arthroplasty and found no significant difference in blood loss or transfusion rates between the two groups [17]. Additionally, a meta-analysis of 34 publications on TXA in total hip arthroplasty failed to identify any statistically significant differences in terms of higher doses, repeat dosing, or variation in dose timing when evaluating total blood loss and transfusion rates [18]. Our findings among patients with extracapsular hip fractures are in line with these previous results. An additional dose of TXA provided no statistically significant differences in blood loss or need for blood transfusions, and there is a possibility that it may not be warranted in clinical practice.

Our study has several limitations. Firstly, it was powered to detect a difference in total blood loss of 341 ml or more between groups, though our results showed a much smaller difference between groups (115 ml). Our review of the existing literature could not elicit an established definition for a clinically meaningful difference in total blood loss in extracapsular hip fracture patients. It is possible that a difference of <341 mL could be considered clinically relevant blood loss, and we were not adequately powered to detect a difference of that size. However, several published meta-analyses and randomized clinical trials on the efficacy of TXA in hip fracture surgery have observed a difference >341 mL in total blood loss between TXA and placebo [14,18-21], which indicates that we were adequately powered to detect an effect size that has been observed in previous studies. Secondly, our study was a retrospective cohort study. A randomized controlled trial could be performed to improve the quality of evidence and eliminate potential confounding effects, such as the difference in BMI across groups that were observed in the present study. In addition, other variables that might play a role in blood loss or transfusion rates should be explored, such as fracture pattern, or other radiographic characteristics.

## Conclusions

To our knowledge, this is the first study to explore the impact of TXA dosing on estimated total blood loss in patients with extracapsular hip fractures. It is also the first study to compare TXA administration at the time of admission alone versus that at the time of admission and again intraoperatively. The results of our study indicate that an additional intraoperative dose may provide no additional benefit. Future randomized clinical trials are warranted to explore the impact of TXA timing and dosing on perioperative blood loss and help define an optimal TXA regimen for extracapsular hip fracture patients.
